# Preparatory graduate professional training in general practice by using the 'experiential learning' framework

**DOI:** 10.1186/s12930-018-0042-1

**Published:** 2018-05-29

**Authors:** Mora Claramita, Fitriana Murriya Ekawati, Aghnaa Gayatri, Wahyudi Istiono, Adi Heru Sutomo, Hari Kusnanto, Mark Alan Graber

**Affiliations:** 1 Department of Medical Education, Faculty of Medicine, Universitas Gadjah Mada (UGM), Radiopoetro Building 6th floor, Jalan Farmako Sekip Utara, Yogyakarta, 55281 Indonesia; 2 Department of Family and Community Medicine, Faculty of Medicine, Universitas Gadjah Mada (UGM), Radiopoetro Building 1st floor, Jalan Farmako Sekip Utara, Yogyakarta, 55281 Indonesia; 3 Department of Family Medicine, Carver College of Medicine, University of Iowa, Iowa City, USA

**Keywords:** General practice/family medicine education and training, Experiential learning, Person-centered care

## Abstract

**Background:**

General practitioners (GPs) in Indonesia are medical doctors without formal graduate professional training. Only recently, graduate general practice (GP) is being introduced to Indonesia. Therefore, it is important to provide a framework to prepare a residency training in general practice part of which is to equip GP graduate doctors to deliver person-centered, comprehensive care in general practice. Experiential learning theory is often used to design workplace-based learning in medical education. The aim of this study was to evaluate a graduate professional training program in general practice based on the ‘experiential learning’ framework.

**Methods:**

This was a pre-posttest study. The participants were 159 GPs who have been practicing for a minimum of 5 years, without formal graduate professional training, from two urban cities of Indonesia (Yogyakarta and Jakarta). A 40-week curriculum called the ‘weekly clinical updates on primary care medicine’ (WCU) was designed, where GPs met with clinical consultants weekly in a class. The participant’s knowledge was assessed with pre-posttests involving 100 written clinical cases in line with each topic in the curriculum. Learning continued with a series of group discussions to gain reflection to reinforce learning.

**Results:**

Participants’ knowledge regarding clinical problems in general practice was moderately increased (*p *< 0.05) after the training from a mean score of 50.64–72.77 (Yogyakarta’s doctors) and 39.37–51.81 (Jakarta’s doctors). Participants were able to reflect on the principles of general practice patient-care. Participants reported satisfaction during the course, and expressed a desire for a formal residency training.

**Conclusions:**

A graduate educational framework for GP based on the ‘experiential learning’ framework in this study could be used to prepare a graduate GP training; it is effective at increasing the comprehension of general practitioners towards better primary care practice.

## Background

‘Family medicine’ is well established in some countries, including in the United States and European countries as a medical specialty which focuses on ‘holistic approach—people-based—comprehensive care’. In commonwealth countries, the term ‘family medicine’ is also known as ‘general practice’ [1]. The World Federation of Medical Education (WFME) introduced three standards of basic medical education (BME), postgraduate medical education (PGME) (which includes family medicine specialization), and continuing medical education (CME) [2]. However, some countries still allow general practitioners (GPs) to work doctors working in a primary care settings without formal graduate professional training in family medicine or general practice [1, 3, 4].

In the era of universal coverage, a paradigm shifting from disease-oriented care to a more person-centered and goal-oriented care is needed. This shift includes the changing of perspectives from fee-for-service to public policy reformation, creating a national health insurance system, making sure all people have equal access to health care) [5]. Those reforms will need leadership including policies and regulations regarding health care services aimed at a more people-centered care. Primary health care should be optimized and prioritized, while family and community empowerment should be encouraged; ancillary workers (e.g. nurses and pharmacists) should work hand in hand with physicians in primary care settings [6]. Therefore, educational strategies should facilitate this movement towards strengthening primary health care. However, specialty training in general practice is still unfamiliar in some countries, including Indonesia. Based on international recommendations, preparation must be made to provide a graduate educational framework for general practice education [1].

Experiential learning theory has been introduced and used for learning at clinical workplaces in medical education worldwide. It was first introduced by Kolb in 1984 and recognizes the importance of ‘reflection’ in the learning process and the need for ‘social context’ to deepen the meaning of learning [7, 8]. The four stages of the learning cycle described in Kolb’s theory starts from ‘concrete experience’ (preferable from real patient-care experiences); this should be followed by ‘reflective observation’ and then ‘abstract conceptualization’; all of these methods apply the process of learning by seeking for information until gaining a complete understanding. Ultimately, the cycle is followed by ‘active experimentation’, to practice and validate the comprehension of the learners. Different ‘learning styles’ have been identified from which learning actions or steps are accentuated after an ‘experience’. The learning tendency may be ‘reflective’ or based on observational methods, more ‘critical thinking’ based on evidences, more ‘experimenting’ through trials, or more ‘accommodating’ by frequently repeating another ‘experience’ [7, 8].

Experiential learning methods have been adopted as learning strategies in medical education for basic and postgraduate medical education [9–16]. Most surgical specialists’ learning styles found were primarily ‘experimenting’ (between theorist and pragmatist), while family medicine or GP residents were found to have a more ‘assimilating’ learning style (between reflector and theorist), whereas anesthesia residents were found to be more ‘accommodating’ (between pragmatist and activist) [9–12]. There is clearly a need of general practice/family medicine graduate programs to participate in the development of better quality health care. General practice demands a highly flexible curriculum with a mix of in person and online courses to provide easier access to continue learning from community-based clinical settings [12].

Much of literatures about ‘experiential learning’ design discuss integrated clinical workplace-based learning at community-based educational settings using reflective journals and collaborative learning as the main strategy to elicit socio-behavioral abilities such as communication skills, leadership and family-care centeredness at primary care settings [13–16]. Two of these publications specifically explained instructional design with lesson plans based on the four stages of experiential learning by Kolb and colleagues, but were intended for undergraduate medical education students [15, 16].

This study aimed to evaluate the use of the four learning cycles in the experiential learning approach to develop and test a more systematic graduate professional training for general practitioners. This study is essential to provide a general practice prototype training framework for doctors who have no formal graduate training in primary care following their undergraduate medical education, but have been working for several years at primary care settings.

## Methods

This is a pre-posttest study followed by a qualitative exploration to evaluate the training approach, using experiential learning cycles, to increase the general practitioners’ knowledge in the area of family medicine and primary care. None of the GPs in this study had any formal graduate professional training in general practice; as graduate training in general practice did not exist in Indonesia at the time of this study.

We developed a 40-week syllabus and lesson plans for a weekly graduate GP educational program, based on the four learning cycles of ‘experiential learning’ theory (‘concrete experiencing’, ‘reviewing and studying’, ‘abstract conceptualizing’ and ‘active experimenting’) [7, 8]. Table 1 describes the syllabus of the graduate family medicine CME program given over 40-weeks namely the *‘*Weekly Clinical Updates on Primary Care Medicine’, which were assisted by clinical consultants from different specialties and general practice team staff-members, all from the Faculty of Medicine, Universitas Gadjah Mada (UGM), Yogyakarta, Indonesia. All GP staff members held academic degrees in medicine (masters, doctorate, and professorship). They had been practicing as generalists for more than a decade at the UGM clinic, but had no formal degree in graduate professional training as a GP (due to the lack of general practice specialty training in Indonesia).

**Table 1:** The 40-week curriculum of “*Weekly Clinical Updates on Primary Care Medicine*” for general practice in this study

Week	Content	Construct of Curriculum in this study Sources: textbooks of general practice [17, 18] reviewed by: a Technical Assistant from World Organization of Family Medicine [19]
0	Pre test	Construct of assessment Source: family practice specialty examination board review [21]
0	Technical meeting on the method of the course, the lesson plan (based on experiential learning cycles), the formation of small group learning community and introducing guidelines for critical appraisals	Foundations of family medicine
1	Promotion and prevention overview (the natural history of illnesses and continuity of care)	
2	General practice principles (the power of better communication skills for better health care services)	
3	‘Ready to work’ (understanding social determinant of health and ‘*bio*-*psycho*-*socio*-*cultural*-*spiritual*’ background)	
4	Prevention of Fe deficiency in young women and pregnancy	Women’s health
5	Prevention of hypertension in pregnant mother	
6	Clinical management of TORCH infection in pregnancy	
7	“Healthy baby—serene mother—happy family” (antenatal care)	
8	Smart patient—proper family planning devices	
9	HPV vaccination and early detection of cervical cancer	
10	Early detection of breast tumor and cancer	
11	Effective management of vaginal discharge	
12	Healthy kid (under five)—adequate nutrition	Child’s health
13	Healthy kid—complete vaccination	
14	Clinical management of dehydration in acute diarrhoea in children	
15	Clinical management of convulsion in children	
16	Clinical management of anxiety and depressions in primary care settings	Mental health
17	Comprehensive management of post-traumatic stress disorder	
18	Early detection and long-term effective treatment of schizophrenia at primary care settings	
19	Understanding epidemiology of mental disorders	
20	Evidence-based practice on ‘headache’	Neurology problems
21	Rational therapy on Bell’s Palsy and other peripheral neurology disorders	
22	Prevention and prompt treatment of STROKE	
23	Comprehensive care for elderly people	Adulthood, elderly and chronic care
24	Clinical management of arthritis	
25	Clinical management of TB patients and minimize the drugs side effects and resistance	
26	Evidence-based practice on asthma and COPD	
27	Screening and managing diabetes type II	
28	Up-to-date of managing diabetics ulcers	
29	Proper nutrition for metabolic syndrome	
30	Effective management of hypertension	
31	Complementary alternative medicine	
32	Evidence-based practice on abdominal pain	Acute care, surgery and infections
33	Are you at risks of prostate hyperplasia or cancer?	
34	Emergency of heart disorders	
35	HIV and voluntary counselling and testing (concern for disadvantage population)	
36	‘5 days’ fever and its differential diagnosis in Indonesian settings	
37	Effective treatment on ‘burn’	
	Early detection on blindness risks (cataract, glaucoma, diabetes retinopathies)	Sensory organs (EYE)
38	Rational therapy on ‘common cold’	Sensory organs (ENT)
39	Early detection on nasopharyngeal cancers	
40	Understanding leprosies for diagnosis and treatment at primary care settings and selection of topical treatment for dermatitis	Sensory organs (skin care)
41	**Post-test**	**Construct of assessment** Source: family practice specialty examination board review [21]

The curriculum of the program was constructed based on textbooks of family medicine/general practice [17, 18] and was reviewed by a technical assistant (TA) from the World Organization of Family Doctors in 2013 [19]. The curriculum was revised based on the comments received from the TA. The main comment was that the content was too clinical and should accommodate more general principles of family medicine. In accordance with this advice, we reworked the first 3 weeks to emphasize the more basic comprehension of family medicine content.

## Subjects

### Subjects

The subjects in this study consisted of convenience samples of 61 GPs from Yogyakarta region (central Java Island) and 98 GPs from Jakarta, Indonesia; who were specifically assigned by their local district health care authorities to do the 40-week graduate course in general practice in 2016–2017. Women predominated our samples (81.97% of Yogya’s and 82.65% of Jakarta’s). The proportion of females in the cohort represents the current mix of Indonesian medical students who are mostly female [20]. Additionally, It is likely that male doctors will continue to seek graduate professional training of medical specialties which are hospital-based, after several years working at *Puskesmas.* Consequently, mid-career doctors at Puskesmas are mostly female [20].

All of the participants are government employees and working at urban sites. The other consideration was the active working period of the participants; they should not be a new employee nor approaching their retirement. The working experiences as general practice of the participants were varied from 8 to 30 years and their ages were between 32 and 55 years old. An informed consent form was completed by all participants before the clinical cases pretest was distributed.

## Instrument

Instruments in this study included: a curriculum and lesson plans of graduate general practice CME program presented in Tables 1 and 2, completed using various methods of instruction. Pre and posttests of clinical cases were written based on Graber’s and Wilbur’s specialty board-review book of family medicine and an examination guide book with typical items used to assess family medicine residents in the US [21]. The items in the board-review book represent family medicine principles, continuity of care across ages, genders and stages of illnesses, written by 40 family medicine specialists as contributors, presented in vignettes/cases followed by several multiple choices. The items in the book have been endorsed as part of preparation for the examination for family medicine residency in the US.

**Table 2:** The lesson plan (instructional design) of each session/week/topic in the “*Weekly Clinical Updates on Primary Care Medicine*” for general practice in this study based on experiential learning cycles [7, 8]

Time	Content	Persons in charge	Experiential learning cycle	Aim	Learning tools
The first 30 min	Presentation of a case based on the topic of the day (an actual patient care)	Primary care physician (1)	‘Concrete experience’ (what was usually done in practice)	To start the learning cycle according to Kolb and colleagues	A medical record based on family medicine principles
The second 30 min	Presentation of a critical appraisals on a publication of *family medicine journals* related to the topic of the case-report	Primary care physician (2) from the same small group learning as physician (1)	‘Reviewing and studying’ (what should be done based on evidences)	To move to the second stage of learning cycle in the experiential learning	Critical appraisals tools and checklist available on the internet (introduced in a workshop of critical appraisals)
The third 30 min	Presentation of a critical appraisals on a publication of *clinical medicine journals* related to the topic of the case-report	Primary care physician (3) from the same small group learning as physician (1)	‘Reviewing and studying’ (what should be done based on evidences)	To move to the second stage of learning cycle in the experiential learning	Critical appraisals tools and checklist available on the internet (introduced in a workshop of critical appraisals)
The fourth 30 min	Feedback and discussion	Clinical teacher who were invited based on the topic of the case-report	‘Abstract conceptualization’	To move to the third stage of learning cycle in the experiential learning	Teacher training on ‘constructive feedback and one-minute preceptor-ship’
The last 30 min	Feedback and discussion	Family medicine teacher from family medicine team	‘Abstract conceptualization’	To move to the third stage of learning cycle in the experiential learning	
Days after	Observation-based learning	Family medicine teacher from Family medicine team	‘Active experimenting’	To move to the last stage of learning cycle in the experiential learning	The one-minute preceptorship
Days after–before another week	Writing a reflection form	All primary care physicians as participants in the WCU course	‘Active experimenting’ (what should be done better next time/plan)	To move to the third stage of learning cycle in the experiential learning	Reflection form based on Gibbs’

The items in the chapters of the Family Medicine Specialist Book review were written and constructed based on the five levels of prevention of primary care principles [21, 22]. Vignettes and cases were written from simple to more complex health problems followed by multiple choice questions. Readers were invited to think through five levels of natural history of each of the diseases selected from simple to the most complicated ones.

Among the total of 30 chapters in the book, the authors of this study selected only ten constructs or clinical topics that represent world-wide illnesses followed with a selection of ten vignettes/cases of each topic (Table 1). The ten vignettes/cases selected consisted of two items of each of the five levels of prevention. For example, for the topic of ‘cardiovascular’, there are two items for primary prevention of cardiovascular diseases (one item for health promotion and one item for specific protection), four items of secondary preventions (two items of early detection and two items of prompt treatment), and finally four items of tertiary prevention (two items of complication-detection and two items of rehabilitation or palliative care). In total there were 100 multiple choice questions pertaining to the ten vignettes/cases. All materials were translated into Bahasa Indonesia and adapted to the Indonesian context and reviewed by specialist consultants and GP staff members from UGM.

The same 100 items applied for the pre and posttests for both study settings. We avoided items which involved advanced clinical laboratory tests or advance pharmacological drugs that could not be done in a *Puskesmas*. The scores from all participants were tallied by a secretary afterwards and sent to the statistician directly, to do the analysis.

The lesson plans as described in Table 2 were designed based on an experiential learning design of the four learning cycles from Kolb [7, 8]. We provided a ‘holistic medical record’ template comprising the bio-psychosocial background of patients and their family to be one of the instruments for the participants in this study [18, 19]. This specific medical record format was used to capture the ‘active experimentation’ of patient-care of the participants based on principles of family medicine, during the first stage of the experiential learning cycle. We also provided guidelines for critical appraisals to help the participants to review several published papers. We promoted the evidence-based practice during the ‘reviewing and studying’ stage of the experiential learning cycle [23]. Teachers in this program who are clinical consultants and team members of GP staff were also trained on the use of ‘one-minute preceptorship’ to provide constructive feedback [24, 25]. Ultimately, ‘abstract conceptualization’ and ‘planning active experimentation for future patients’; the last two stages of the experiential learning cycle were guided by a written reflection and a series of group discussions with the participants after each topic was presented [25].

Group discussions were conducted 5 times with participants from Yogyakarta and 10 times with Jakarta’s GPs. The main guiding questions were, “Tell us a story about a patient that you most remember,” (the identity of the patients remained confidential) and “How do you reflect on the patient-centered clinical care?” We adapted the generic rules of the Balint group discussions: (1) no interruption from the start until the end of a story that is told by a doctor, (2) no judgment based on doctors’ ability, (3) no suggestions or comments from other participants—only questions for clarification, (4) if there is any reflection it will come from the presenter of the story by her/him selves or other doctors who listen to the story, while (5) the identity of all persons remained confidential, (6) nobody had access to a paper, or pen, or mobile phone, or a laptop, and (7) the recording is based on an informed-consent process, e.g. for this study’s purposes [26, 27].

## Procedures

The pretest was administered before the first learning session. The pretest was followed by a technical meeting session where the large group of participants was divided into small groups, each consisting of ten physicians, who worked together for each of the assignments. We also provided a rigorous explanation of the curriculum (Table 1), the lesson plans (Table 2) and other learning tools [23–25]. We prepared the participants to learn with the following strategies: (1) by experiential learning cycle basis, (2) to work together in a collaborative small team group, (3) to regularly appraise literatures, (4) to do series of individual reflections, (5) to do group discussions, and (6) to be open to any feedback from the peers and clinical consultants. The key message was ‘continuous learning’.

After each technical meeting session, the first learning session was implemented. One of the participants presented an actual patient-care case, using a ‘holistic and comprehensive medical record’ format which was designed specifically for family medicine doctors that includes bio-psycho-social-cultural aspects, continuity of care and comprehensive care [18, 19]. The main idea was used to start the learning process with an ‘actual experience’ of the experiential learning cycle [7, 8]. Afterwards, another two participants within the same small group of learning presented a critical appraisal of a journal article (clinical and family medicine articles), which were closely related to the case presented in advance. The aim of this critical appraisal session was to facilitate the participants receptiveness to evidence-based practices and included in the ‘reviewing and studying’ of the experiential learning cycle [7, 8]. Each of the participants who presented cases and critical appraisals wrote at least five questions to be presented to the class and clinical consultants who attended the session. The feedback session was conducted by the clinical consultants and GP staff members. The clinical consultants addressed each of the questions posed by participants, teaching general rules and applying the ‘one-minute preceptor’ method in this feedback session [24, 25]. Sometimes the consultant needed to describe an illustration with a slide or two, but this media was not considered ‘lecturing’ time. Questions from participants guided the discussion/explanations. The GP staff members acted as moderators in this session and continued to provide feedback appropriate to the subject of family medicine/general practice. The feedback session was intended to facilitate ‘abstract conceptualization’ within the experiential learning cycle.

Considering the limited time available to GPs, we scheduled 2.5–3 h/week to do each session. Sessions were held on Saturdays at the Faculty of Medicine UGM, when the patient-care schedules were minimum. The UGM staff members from the GP team then visited each group of participants to be a facilitator of the ‘active experimentation’ stage of the experiential learning methods. Finally, each participant wrote a reflection and prepared for another topic for the next week’s session. With these goals, the learning process would be on-going for the entire week. At the end of the 40 weeks of sessions, a post test was administered and series of group discussions were conducted.

## Analysis

The authors did a paired sample and independent sample t test for the analysis of the clinical cases’ items [28] and also a qualitative open coding for the results of each of the focus group discussions in each topic/lesson [28]. The participants’ comments on the course-construct were analyzed until saturation of data was gained [28]. We categorized each of lessons learned based on each topic of the course.

## Results

The t test paired sample proved to be significant, comparing the results from before and after the 40-weeks of ‘weekly clinical update’ sessions using experiential learning design intervention (*p *< 0.050) as presented in Table 3. Yogyakarta’s doctors increased their mean scores from 50.64 (pretest) to 72.77 (posttest) and Jakarta’s doctors increased from 39.37 (pretest) to 51.81 (posttest). The progress of learning was indicated by the Delta scores of Yogyakarta’s doctors (22.13) and Jakarta’s doctors (12. 44). The approach of training in this study using four cycles of experiential learning was demonstrated to significantly increase the level of knowledge of the doctors at both cities (Table 3).

**Table 3:** The results of pre-posttest of the participants in this study

Regions	Doctors	Pre-test scores					Post-test scores					Mean ∆ post–pre scores (95% CI)
	N	Lowest	Highest	Mean (95% CI)	Median	SD	Lowest	Highest	Mean (95% CI)	Median	SD	
Y (overall)	61	28	64	50.64 (48.25–53.03)	52	9.54	52	86	72.77 (70.71–74.83)	72	8.21	22.13 (19.36–24.90)
Y (female)	50 (81.97%)	28	64	49.96 (47.31–52.61)	51	9.56	52	86	72.20 (69.88–74.52)	72	8.36	22.24 (19.09–25.39)
Y (male)	11 (18.03%)	35	64	53.73 (48.25–59.21)	55	9.27	61	83	75.36 (71.05–79.67)	80	7.30	21.64 (15.84–27.44)
J (overall)	98	10	58	39.37 (37.73–41.01)	40.5	8.27	38	65	51.81 (50.50–53.12)	53	6.63	12.44 (10.49–14.39)
J (female)	81 (82.65%)	10	58	39,64 (37,87–41,41)	42	8.12	39	65	53.00 (51.70–54.30)	53	5.95	13.36 (11.41–15.31)
J (male)	17 (17.35%)	10	50	38.06 (33.76–42.36)	40	9.05	38	61	46.12 (42.84–49.40)	47	6.90	8.06 (2.08–14.04)

Regions	*p* value (paired t test/independent t test)											
	Pre vs post	Pre-test					Post-test					
		Y (A) vs J (A)	Y (F) vs J (F)	Y (M) vs J (M)	Y (F) vs Y (M)	J (F) vs J (M)	Y (A) vs J (A)	Y (F) vs J (F)	Y (M) vs J (M)	Y (F) vs Y (M)	J (F) vs J (M)	
Y (overall)	0.000	0.000	0.000	0.000	0.244	0.512	0.000	0.000	0.000	0.223	0.001	
Y (female)	0.000											
Y (male)	0.000											
J (overall)	0.000	**∆ Post-test–pre-test**										
J (female)	0.000	**Y (A) vs J (A)**	**Y (F)vs J (F)**	**Y (M) vs J (M)**	**Y (F) vs Y (M)**	**J (F) vs J (M)**						
J (male)	0.018	0.000	0.000	0.004	0.860	0.115						

*Y* Yogyakarta, *J* Jakarta, *A* overall, *F* female, *M* male

Overall, using t test independent samples, Yogyakarta’s doctors were found to have higher scores than the Jakarta’s doctors in pre and posttests and also Delta scores (*p *< 0.05). As noted the samples were predominantly women, and when we analyzed the data the Yogyakarta’s female doctors were found to have higher scores than Jakarta’s female doctors in pre, posttests and Delta scores (*p *< 0.05). However, there were no significant differences between females and males in each of the two cities, for pre-test and posttest and the Delta scores (*p *> *0*.05), except the posttest of Jakarta’s’ female and male doctors (*p *< 0.05) (Table 3).

The GPs commented about the course in this study suggesting a more longitudinal—a yearlong course, integrated graduate course where the GPs staff-members and specialists sit in the same row to provide feedback for the participants. Also, it was suggested that it should be the participants who lead the discussions through the questions and not the specialist. Some GPs’ opinions are as follows:

*“Critical appraisals are difficult, but it opened new information which I would never imagine, medical evidences change very fast over the time.”*

*“I already work as a GP for more than 30* *years and I am about to retire. But this course is what we actually need and I would like to study further into a formal vocational training of family medicine.”*

*“We need the clinical part of patient care training more in this course.”*

*“The specialists should provide ‘referral back’ letter to us. They rarely do that. We need to learn from our referred cases and we will be with the patients whenever they are.”*

The results of 15 group discussion sessions are presented in Table 4. Across all topics provided in the course, there were some topics which were not addressed by the participants in this study, e.g. ‘sensory organs topics’ and ‘child health’. This phenomenon may be due to limited time for the group discussions in this study, or it may be that GPs in this study were not used to dealing with those specific problems due to lack of equipment for sensory organs’ diagnosis and treatment at the primary care settings, or specialists and midwives may have taken care of the problem adequately (e.g. for the child health).

**Table 4:** Results of qualitative open-coding analysis in this study

Categories	Quotations
Foundation of family medicine	
The comprehension of the importance of evidence-based practice	*“Looking for an evidence*-*based practice is possible for a family physician because the literature are always change throughout the time. The literature of family medicine also strongly suggested that we should keep up to date with the newest evidences. I think it is good if we build a network among us (the GPs), so that we could discuss any evidences”*
The use of complementary alternative medicine	*“I had a patient with cerebral palsy and she lives at home with her mother who already very old. She did not have any access to hospital and then a nearby midwives was asking for my help. So I initiated to do a home visit with my nurse. The patient could not move and having very severe infection of ‘decubitus’ and also on her vagina, the worst wound you may imagine. I then remembered from the weekly clinical update that we may use honey. So I tried. I clear her wounds each day, slowly, and surprisingly it worked out very well. Just a regular honey!”*
	*“My patient had a diabetic ulcer and I referred him to the hospital and the surgeon decided to do ‘amputation’. The patient refused. He came back to me and insisted that he only wanted me to care for him. I was puzzled. Then I remember from the Weekly update, the appraisals about honey and the discussion with the faculty surgeon during that appraisal session. I did an informed consent with the patient. And also that he must obey the regular diabetic treatment that he has. So I started to debridement the wound and carefully use regular honey. Surprisingly, after e period, the wound healed, he could walk normally and now he keeps healthier life”*
The importance of home-visits and understanding a family	*“As a GP, we could also do a home*-*care and home*-*visits, so that we understand the family context of an elderly. There is new financial system at the primary care centers, I am sure we could manage it to provide extra incentives for health professionals who do home care and home visits”*
The comprehension on family and individual life cycle in regards to individual illnesses	*“Thank you for giving me an opportunity to reflect on elderly patient* – *care with many complex problems. I knew now the cycle of life and cycle of a family so I understand that it is also difficult for her son (who also has a family and adolescence teenagers) to take care of their parents at home. It is a complexity of life that we, as a family physician should understand better”*
Initiated community group learning	*“Starting to care for chronic illnesses like hypertension and diabetes mellitus based on the national insurance program, we formed a community health group meeting; which ultimately self*-*funded and self*-*regulated. It is amazing to see that the elderly people initiate to have periodic meetings, recreation…”*
Closer steps to patient centred care	*“I understand that listening, is very important and we may come to different diagnosis and more correct treatment after deeper listening”*
	*“Now I know that we should ‘well*-*prepared’ the patient before they referred to a hospital, so sufficient information is highly important for the patient to understand what specialists may do in hospitals and how they could discuss with them”*
	*“As a fresh graduate doctor, twenty years ago, I worked at a remote area. There was an abortion case and I was following a correct procedure and then referred the patient to a hospital. However, I might not able to do an adequate communication with the family (since I did not know the technique until I joined this course) so I was on a ‘trial’ in front of a ‘community court’. No lawyer or advocate stood at my side. The husband was misunderstood and blamed me causing his loss of a child. Fortunately, the community trusted my explanation, and I knew that the husband had a several records of misbehave in that community. However, I should not only thinking and performing correct medical procedures, I must communicate with the patient and their family better”*
Women’s health	“*There was a teenager who was unmarried and pregnant without a husband. She was so depressed and so I have to conduct a family meeting. Her parents; respectable couple in the neighborhood, could not accept the condition and forced to do an abortion. I explained the healthy condition of their daughter and future grandson and so I have taken initiative to assist the family, day by day, week by week, up to years later to going through every stage of a family crisis. Now they are happy and the daughter could continue her education* via *distance learning program. Now I know that what I did was a part of being family doctor and I am very happy for that”*
Child’s health	No discussion on child health problems
Mental health	*“The WHO recommended continuity of care for mentally ill patients and shifted from hospital based into community based care. The recommendation has consequences of strengthening primary care team at primary care settings, empowering the family, educating the family and community, and researches in the area of mental illness. Our community clinic has been trying to implement these recommendations* via *home visits, psycho*-*education for the family, the formation of cadre for mental illness, group therapy for the patients and also for the family, all in a program called Health Village Mentally Resilience. We also should work together with all health professionals and hospital, to provide two*-*way comprehensive care towards patient*-*centered care. There are so many mental disorders at the community settings”*
Neurology problems	*“I was on duty in a rural area and a patient came back to our clinic. He suddenly could not talk (a young man), has a very weird movement on his extremities. It was not a stroke, but what was it? His father kept talking to me about bad spirit that his neighbor had sent to his family”* *“While his father was talking* - *I had to extremely divide my attention with thinking to a more logical way. I did careful history taking and the patient apparently has consume the metoclopramide. I remember the possibility of allergic and extrapyramidal effects. Fortunately in the clinic we had the antidote that was ‘trihexyphenidil’. I saved the patient and his family was grateful because I won over the bad spirit. Then I explained to the family what happened and he should avoid any metoclopramide”*
Adulthood, elderly and chronic care	
The idea to optimize the home-institution for elderly people which was unfamiliar for the context of this study	*“I think it is the time to optimize the home*-*institution for elderly people. Usually we perceived that an elderly home is for neglected elderly. We should have a new perception now that an elderly home is for any elderly who need it. And elderly home could be the best place for them to have a social relationship, to talk, chat, and play games, because elderly needs others to share stories”*
	*“I had a patient with diabetes mellitus type II, chronic. She was often come to my clinic and I already explain anything related to prevention and treatment of diabetes and its complication. She refused any referral to hospitals and she would only visit me. I know her condition got worse and worse and I motivated her to use insulin or to visit hospital. One day she never come again, I know from her neighbor that she died. I should regularly visit her at her home, I regret I never do that…”*
Acute care, surgery and infections	
Emergency in baby delivery	*“When I was on duty on a remote area of Indonesia, there was an emergency baby delivery that we should be used a vacuum. The baby were twins. It was just me and one very young midwife and no hospital. We did all the procedures correct, but the vacuum was not in good function. The mother was safe, but the twins were died immediately after born. I should have re check all the emergency equipment regularly especially when no other health care settings is around”*
Emergency in shock syndrome	*“Once there was a patient with a somewhat medium late allergy reaction came back to our clinic, one hour after a molar excision. The dentist already went home, it was after the working hours. I was lucky that the nurses were so helpful in assisting me not to panic and I realize that I have to check the adrenalin and all other emergency drugs and procedures regularly; no place for expires. Thanks God we saved that patient”*
HIV problems	*“I have a patient, he is a teenager and having the HIV. He came to me in his worst condition with candida all over his body. He seemed like a ‘tree’ than a ‘human’. But I tried to communicate with him as a friend. He used to text me until he trusted me to bring his partner to Puskesmas. The partner was agree to do a counseling prior to an HIV test. I also communicated with them that it is important for their parents to know. For this special patient, holistic and comprehensive care as I learned in this course is certainly needed. I also learned on how to do steps of family intervention in a more effective way”*
Sensory organs (eye)	No discussion on sensory organs problems
Sensory organs (ENT)	
Sensory organs (skin)	

## Discussion

To the best of our knowledge, this is the first study which reports the use of experiential learning theory framework for postgraduate medical education. It was designed specifically for preparing professional training in general practice and to stimulate more student-centered learning. Other studies using experiential learning theory emphasized residents’ learning style but did not touch on the instructional design of using the four learning cycles in the experiential learning theory to promote a more independent and lifelong learning [9–12]. The ‘experiential learning’ framework in this study provides significant examples of stimulation to the GPs to have more self-directed learning strategies, driving the GPs into a more active and reflective learning. It presents the challenges and opportunities of better patient-care services in the universal coverage era and also illustrates a more collaborative relationship between the general practice staff members, clinical consultants and many other stakeholders who supported the innovation of medical education in this study. Therefore, although this study was done in an Indonesian context where graduate training for GPs does not exist, the ‘experiential learning’ framework in this study may be adapted to a context where postgraduate program has been implemented for many years.

In the first ‘concrete experience’ stage of experiential learning cycle during the course, we did not have much difficulty, since physicians in this study are used to reporting any patient-care they did at primary care settings. However, the form of ‘holistic and comprehensive medical record’ formats we used throughout the training were quite challenging involving not only focusing on the illness but also being attentive towards patient-centered care. Instead of focusing only on certain diseases, GPs in this study were also directed towards patient-centered care, family focus and community oriented clinical thinking; concepts which were lacking during their undergraduate medical education [17, 18].

Many of the participants wondered whether they should do a patient-centered care approach for every patient, because they perceived that they won’t have enough time. The discussion on the limited time for patient-care services revealed that it required usually less than 5 min of patient-care services for each patient at primary care centers in this study, which is not different than in the hospital settings in a previous study [29]. The general practice staff members as facilitators in this study emphasized the discussion on the use of an ‘appointment system’, which most of general practitioners in this study already did for, but for only the antenatal care and elderly care patients. Therefore, primary care centers in this study may have separated the intake process of the quick encounter for ‘quick care’ and the others as ‘appointment encounters’ to provide better health care services as recommended in the literature [1].

For the second stage of the learning cycle, which is ‘reviewing and studying’, it was considered difficult as perceived by most of participants in this study due to the English language and unfamiliarity with appraising medical literature in their daily practice. The participants were used to listening to lectures or reading local protocols on certain diseases. There were arguments from the clinical consultants whether this stage of ‘journal reading’ is necessary. However, after several topics, the participants felt the benefits of the ‘journal appraisals’ to understand other ‘views’ and scientific reasons behind a basic patient-care guideline or protocols that they usually use in daily practice. Participants’ five questions posed to the clinical consultant at the end of their presentations had successfully directed the discussions into mutual dialog instead of only passively absorbing the information.

The third stage of experiential learning, which is ‘abstract conceptualization’ started with constructive feedback from both clinical consultants and a GP staff member in meaningful discussions. This stage was challenging to always refer to GP principles and not to get overwhelmed with hospital-style patient-care management. Although clinical simulation using mannequins were sometimes performed during this learning stage, the GP staff members aimed to direct the discussions back to the importance of promotion and prevention for certain age groups and different genders and emphasized the undifferentiated cases [17, 18]. Important principles of GP were the ‘watchful waiting’, to think and to observe together with the patients, and to offer adequate information in order to make better clinical decision-making. This program also provided examples of communication and collaborative practice with different levels of health professionals.

The last learning cycle of ‘active experimenting’ was performed in this study and was facilitated by a reflection-form to guide the participants to make a plan for present and future learning. The discussions also served as an effort of trying to listen to physicians’ stories and reflecting on future concerns for patient-care. The GPs in this study completed their learning cycle with planning better services in the future.

There were only a few questions left unanswered by the clinical consultants in the course, for example dealing with lack of time and facilities at primary care centers. However, along with the gate keeper system in the universal coverage era in the context of this study, there is a national accreditation system of the primary care clinical settings in Indonesia which will drive the improved maintenance and increasing quality of primary care services and facilities in the near future [3].

As the early stage of a period of universal coverage changes in the context of this study, health professionals and patients may not yet receive the support and care they deserve, but the progressive approach of education and incrementally better health care services can help. This kind of discussion may ultimately lead to alternative solutions coming from the participants. During this course, the GPs learned to do maximum prevention for the patients, including reducing medication complications by using better quality drugs instead of low level quality of drugs for merely ‘cost-saving’ purposes.

However, we admit that interprofessional collaborative practice was less emphasized in this course and should be better articulated in the future. By improved team work of different health professionals, patient care services may become more effective and efficient [30, 31]. Interprofessional collaborative practice should also emphasize not only curative medicine, but most importantly prevention. It has been a known fact that in order for primary care to function effectively and efficiently, a multidisciplinary team of both clinical and non-clinical professionals must be formed and fully function interprofessionally [30, 31].

## Strengths and limitations

This study used GP samples from Yogyakarta and Jakarta provinces, who consisted of public servant doctors in public primary care clinics. The results should be interpreted in appropriate contexts. However, the combination of quantitative data analysis and in-depth qualitative exploration strengthened the data saturation and interpretation.

This study should be continued to include a formative and summative part of clinical bed site teaching when a formal graduate study program is legally established. In the preparation of providing a framework of graduate educational training for general practice in this study, we succeeded in shifting the way of thinking of the existing GPs from disease centered into a more patient-centered care, appraising more evidence-based practice, and learning in two-way dialogues with other medical specialists. The ultimate accomplishment was a substantial contribution of many clinical departments and stakeholders within a year-long program of GP graduate training. These lessons learned about preparing a graduate professional GP educational framework in this study expectantly could be used for many other countries.

We noticed that the Yogyakarta group of doctors demonstrated a higher level of learning than the Jakarta group of GPs. This phenomenon could have happened due to several reasons such as better learning environment in Yogyakarta, which is well-known as a ‘student city’ in Indonesia, more conducive learning process, and more receptive participants, compared to a metropolitan city of Jakarta. The ‘weekly clinical updates of primary care’ had been conducted in Yogyakarta, every year, in the 5 years prior to this study, so the Yogyakarta doctors may have been familiar with the learning strategy of this program. But none of participants had access to the information to be tested in advance. The program was completed in 40-weeks of sessions and the assessment items were re-edited every year so it is almost impossible to memorize the content.

## Conclusion

This study demonstrated that using the educational framework based on the ‘experiential learning cycle’ significantly increased participants’ knowledge in the context of graduate general practice. The group discussions revealed participants’ deep reflections and future plans concerning better patient care services. Any future effort of establishing a preparatory graduate course of general practice may use the framework provided in this study.
